# Long-Term Cryopreservation of Nasal Polyp Tissue in a Biobank for the Isolation and Culture of Primary Epithelial Cells

**DOI:** 10.3390/ijms24076383

**Published:** 2023-03-28

**Authors:** Jonghui Kim, Karla Hegener, Claudia Hagedorn, Kaschin Jamal Jameel, Daniel Weidinger, Inga Marte Charlott Seuthe, Sabine Eichhorn, Florian Kreppel, Jürgen Knobloch, Jonas Jae-Hyun Park

**Affiliations:** 1 Department of Oto-Rhino-Laryngology—Head and Neck Surgery, St.-Josefs-Hospital Hagen, University of Witten/Herdecke, D-58097 Hagen, Germany; 2 Chair of Biochemistry and Molecular Medicine, Center for Biomedical Education and Research, School of Life Sciences (ZBAF), University of Witten/Herdecke, D-58453 Witten, Germany; 3 Medical Clinic III for Pneumology, Allergology and Sleep Medicine, Bergmannsheil University Hospital, Ruhr-University Bochum, D-44789 Bochum, Germany

**Keywords:** cryopreservation, nasal polyps, CRSwNP, epithelial cells, biobank

## Abstract

Epithelial cells may play an important role in the pathologic process of chronic rhinosinusitis with nasal polyps. Therefore, providing epithelial cells from a biobank could greatly contribute to further research. In the present work, the isolation of epithelial cells from long-term cryopreserved tissue is demonstrated. Polyp tissues were cryopreserved in a commercially available freezing medium with dimethyl sulfoxide and stored in liquid nitrogen. The outgrowth and proliferation of epithelial cells from cryopreserved tissue were evaluated and compared to that of fresh tissue. Flow cytometric analysis with anti-cytokeratin, anti-p63, and anti-Ki-67 was performed to identify epithelial cells and determine differentiation and proliferation. A functionality test was performed by determining type 2–relevant proteins, representatively thymic stromal lymphopoietin (TSLP) and periostin, using ELISA. Primary epithelial cells could be isolated from cryopreserved tissues. Cells from cryopreserved tissues showed comparable outgrowth and proliferation to that of fresh tissue. Isolated epithelial cells showed high cytokeratin, p63, and Ki-67 expression and secreted TSLP and periostin. In the present study, a method for long-term cryopreservation of polyp tissue was established, thereby enabling the isolation and cell culture of primary cell culture at a later time. Epithelial cell availability should be greatly improved by including this method in a biobank.

## 1. Introduction

Chronic rhinosinusitis with nasal polyps (CRSwNP) is characterized in western countries as a type 2 inflammatory disease involving immune cells such as type 2 innate lymphoid cells (ILC2), eosinophils, and mast cells [1]. CRSwNP is a heterogeneous disease, and much evidence suggests further heterogeneity even within the patient population with typical features of type 2 inflammation, such as different expressions of biomarkers and individual differences in response to biological therapy [2,3,4,5]. Therefore, a biobank must be established to enable comprehensive investigations for endotyping with large patient populations.

Epithelial dysfunction is an important pathological process in CRSwNP. Epithelial cells are not only regarded as a barrier against pathogens and external influences but also play a role in the pathomechanism that produces pro-inflammatory cytokines, such as thymic stromal lymphopoietin (TSLP), interleukin-25 (IL-25), and interleukin-33 (IL-33), and trigger a reaction cascade via ILC2 cells [6,7,8]. In addition, an abnormal process, a so-called remodeling, takes place in epithelial cells in patients with CRSwNP, such as epithelial-mesenchymal transition, basal cell hyperplasia, and reduced cell diversity [9,10]. However, the mechanism of dysregulation in epithelial cells, the interaction of epithelial cells with other cell types, and the response of epithelial cells to cytokines are largely unknown. These questions could be well investigated under experimental conditions with cell culture and co-culture with other cell types.

Biobanking of epithelial cells may allow better availability of epithelial cells for further study. With classical methods, epithelial cells were first isolated on the day of surgery; then, the isolated cells were cryopreserved when the number of cells had increased to some extent [11,12,13,14]. This immediate work process affects the flexibility and potentially complicates the biobanking of epithelial cells. The present work demonstrates the long-term cryopreservation of nasal polyp tissue and the isolation of epithelial cells from the cryopreserved tissue.

## 2. Results

### 2.1. Isolation of Epithelial Cells from Cryopreserved Nasal Polyp Tissues

**Figure 1:**
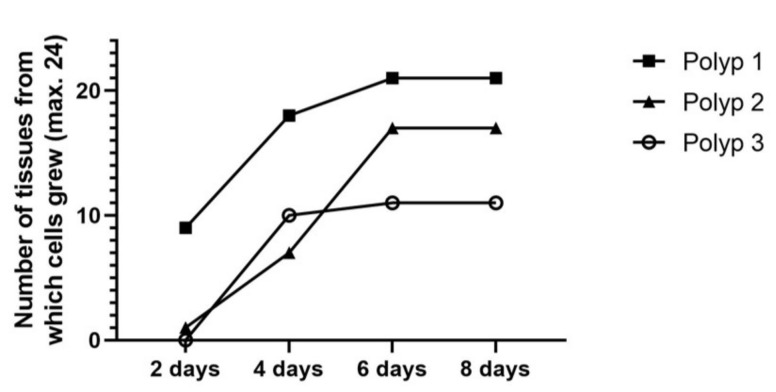
Cumulative number of tissue pieces from which cells grew out. Tissues from 3 patients were taken for the isolation of epithelial cells. Per sample, 24 small-cut pieces of tissue from each patient were used for the outgrowth of epithelial cells. The cumulative number of tissue samples from which cells grew out was assessed every 2 days. Outgrowth of 9, 18, 21, and 21 (Polyp 1); of 1, 7, 17, and 17 (Polyp 2); and of 0, 10, 11, and 11 (Polyp 3) of the 24 small-cut tissue pieces was noted on Days 2, 4, 6, and 8, respectively.

**Figure 2:**
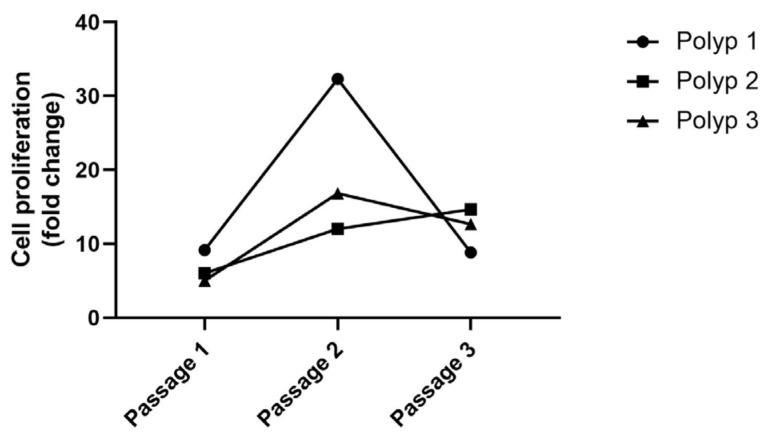
Fold change in cell number up to the 3rd passage. At the beginning of every passage, 30,000 cells were seeded. The number of total cells was counted after 6 days. The epithelial cells isolated from cryopreserved tissues showed a good proliferation rate up to the 3rd passage, approximately 5- to 32-fold. On day 6 of each passage, approximately 275,000, 970,500, and 264,000 cells were counted from Polyp 1; approximately 180,000, 360,000, and 440,000 cells from Polyp 2; and approximately 150,000, 505,000, and 380,000 cells from Polyp 3.

Outgrowth was evaluated from the tissue samples of 3 patients (Polyps 1–3), which were cryopreserved for 262, 196, and 191 days, respectively (Table 1). It was possible to isolate epithelial cells from all 3 long-term cryopreserved tissue samples. Per sample, 24 small tissue pieces (1–1.5 mm in diameter) were cultured to isolate epithelial cells by outgrowth. Of 24 small-cut pieces, 21 (Polyp 1), 17 (Polyp 2), and 11 (Polyp 3), respectively, showed the outgrowth of epithelial cells up to day 8 (detailed data in Figure 1). On day 8 of outgrowth, 140,000 (Polyp 1); 725,000 (Polyp 2); and 55,000 (Polyp 3) cells were collected, respectively. The epithelial cells showed an excellent proliferation rate of approximately 5- to 32-fold up to the 3rd passage (Figure 2).

### 2.2. Comparison of Both Outgrowth and Proliferation Rate of the Epithelial Cells from Fresh Tissue and from Cryopreserved Tissue

**Figure 3:**
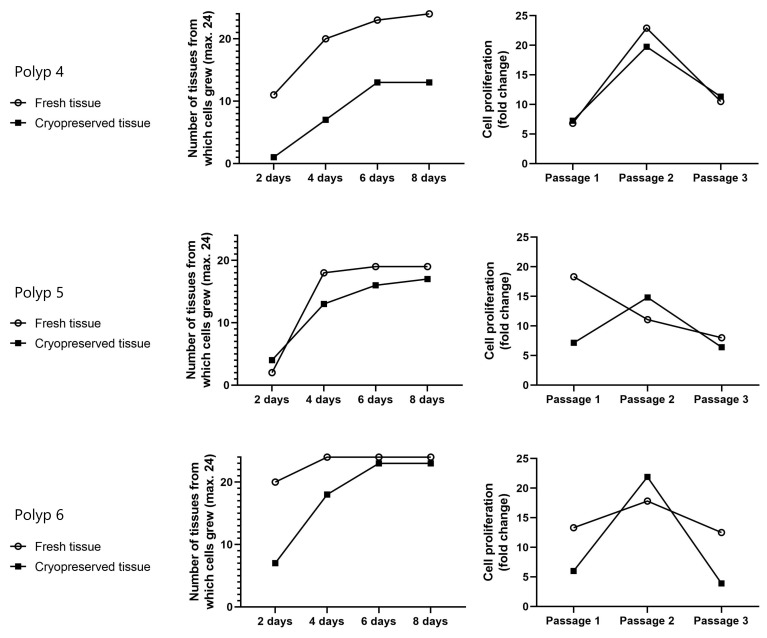
Outgrowth and proliferation of epithelial cells from 3 fresh tissues and their cryopreserved tissues (Polyp 4, Polyp 5, and Polyp 6) were compared. Tissue was cut into small pieces, and 24 small tissue pieces per sample were cultured for the isolation of epithelial cells by outgrowth. The outgrowth was assessed every 2 days for 8 days. To evaluate the proliferation, cells were transferred to a 6-well plate at 5000 cells per well (total 30,000 cells) on day 8 of outgrowth, and the number of cells from 6 wells together was counted every 6 days until the 3rd passage. See Appendix A for detailed data.

**Figure 4:**
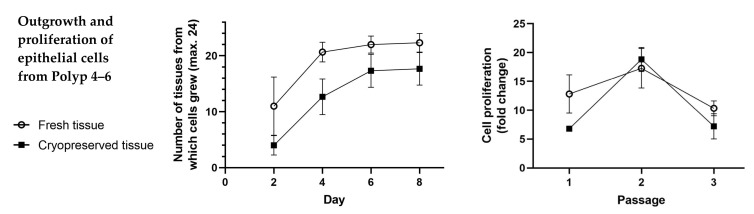
Statistic evaluation of overall outgrowth and proliferation of Polyps 4–6. The outgrowth rate of epithelial cells from fresh tissue was, on average, higher than that of epithelial cells from cryopreserved tissue at any time point (**left**). However, the differences were not significant (*p* > 0.05 on day 2, day 4, day 6, and day 8). Proliferation rates were not significantly different up to the 3rd passage (**right**) (*p* > 0.05 at passage 1, passage 2, and passage 3).

The outgrowth and proliferation of epithelial cells from 3 other fresh tissues and their cryopreserved tissues (Polyps 4–6) were compared. Epithelial cells could be isolated from both fresh tissue and cryopreserved tissue. Each tissue showed a different outgrowth and proliferation rate (Figure 3). Data analysis showed no significant difference in the outgrowth and proliferation rate of the epithelial cells from fresh tissues compared to those from cryopreserved tissues (*p* > 0.05 at all times) (Figure 4).

### 2.3. Flow Cytometric Analysis to Identify Epithelial Cells and Evaluate Differentiation and Proliferation

**Figure 5:**
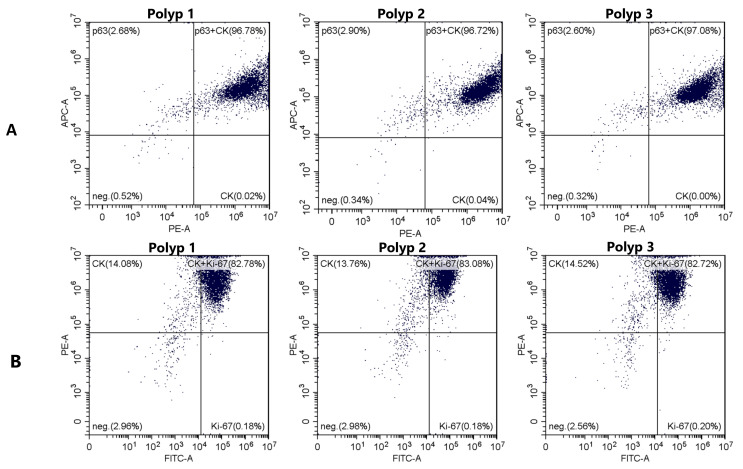
Flow cytometry analysis of cytokeratin, p63, and Ki-67 expression in the isolated cells from 3 tissue samples. (**A**): The relative fluorescence intensity due to CK (PE) and p63 (APC) is plotted on the log scale x- and y-axis, respectively. The proportion of simultaneously positive p63 and CK was 96.86% ± 0.1114%, indicating that the isolated cells are undifferentiated epithelial cells. (**B**): The relative fluorescence intensity due to Ki-67 (FITC) and CK (PE) is plotted on the log scale x- and y-axis, respectively. Most of the cells were Ki-67 positive (82.86% ± 0.1114%) and are, therefore, in a proliferative cell cycle, which reflects good proliferation of the cells. CK: cytokeratin.

The proportion of simultaneously positive p63 and cytokeratin (CK) was 96.86% ± 0.1114%, indicating that the isolated cells were mostly undifferentiated epithelial cells (Figure 5A). In addition, the majority of cells were Ki-67 positive (82.86% ± 0.1114%, Figure 5B), which reflects the good proliferation of the epithelial cells (Figure 4).

### 2.4. Functionality Test by Determining Type 2–Relevant Proteins Using Enzyme-Linked Immunosorbent Assay (ELISA)

**Figure 6:**
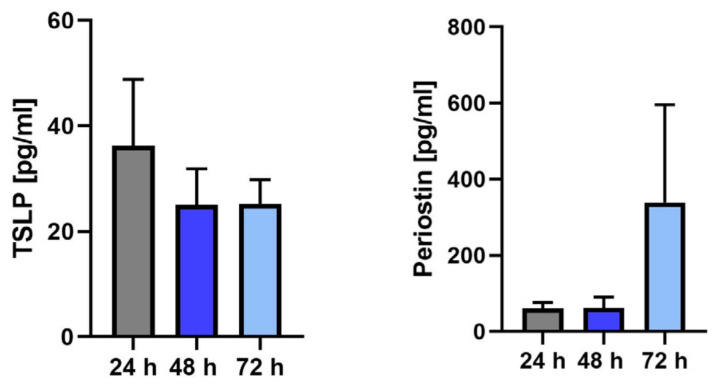
The function of epithelial cells from cryopreserved tissues was assessed by the production of typical cytokines and proteins of type 2 inflammation, representatively TSLP and periostin. The cells were able to produce TSLP and periostin, demonstrating sufficient functioning of the epithelial cells for further study. TSLP concentration (n = 3): 36.22 ± 12.62 pg/mL in 24 h; 24.96 ± 6.917 pg/mL in 48 h; and 25.22 ± 4.619 pg/mL in 72 h. Periostin concentration (n = 3): 60.45 ± 15.70 pg/mL in 24 h; 62.10 ± 28.14 pg/mL in 48 h; and 338.7 ± 256.9 pg/mL in 72 h.

The function of epithelial cells was assessed by determining the production, with isolated cells from cryopreserved tissues (Polyps 3–5), of a pro-inflammatory cytokine and protein produced by the epithelium in type 2 disease, representatively TSLP and periostin. The cells were able to produce TSLP and periostin (Figure 6), demonstrating the functional activity of the epithelial cells and making them suitable for further study.

## 3. Discussion

Establishing a biobank to collect patient materials is beneficial for further comprehensive investigations to clarify the underlying pathomechanism in CRSwNP. Some studies have mentioned the supply of patient materials, such as blood and polyp tissue, from their biobank, without specifying the material treatment for preservation [16,17,18,19,20]. With preserved tissues and blood, immunohistological [16,17] and molecular biological [18,19] investigations could be performed. The cryopreservation of primary epithelial cells in a biobank is principally possible [20]; however, some difficulties of the classic method must be overcome. In particular, the isolation of epithelial cells has to take place promptly on the day of the harvest of tissues, and cells should be cryopreserved within a few days after cultivation [11,12,13,14]. Such a process for days impairs flexibility and costs time to preserve cells, potentially complicating the biobanking of epithelial cells.

Our cryopreservation method with a commercially available freezing medium overcomes this problem. The tissue can be rapidly processed and long-term cryopreserved. The cryopreserved tissues showed a good outgrowth and proliferation of epithelial cells, with a 100% success rate (Figure 1, Figure 2, Figure 3 and Figure 4) but without significant differences from those from fresh tissue (Figure 4). The isolated epithelial cells from cryopreserved tissues showed high cytokeratin, p63, and Ki-67 positivity (Figure 5) and produced TSLP and periostin (Figure 6), indicating no qualitative differences from the epithelial cells from fresh tissue [15].

In the present work, a method of long-term cryopreservation of the polyp tissue for a biobank has been established, enabling a primary cell culture of epithelial cells from a cryopreserved nasal polyp at any later time. Epithelial cell availability could be greatly improved by including this method in a biobank. In further investigations, the feasibility of isolating other cell types from the cryopreserved nasal polyp tissue, such as fibroblasts and immune cells, should be evaluated.

## 4. Materials and Methods

### 4.1. Ethics Statement and Patient Materials

This study was performed according to the ethical standards of the declaration of Helsinki and following approval of the ethical committees of the University Witten/Herdecke (No. 209/2020). Donors gave their informed consent for their nasal polyp tissue to be used for preservation and analysis for research purposes. Nasal polyps obtained from patients with CRSwNP undergoing endoscopic endonasal sinus surgery are the source of the tissue material. Tissues from 6 patients were included in this study (Table 1). The data from Polyps 4–6 were already used in our previous work (named Polyp 1, Polyp 2, and Polyp 3, respectively, in our previous work) to demonstrate the isolation of epithelial cells from fresh tissue [15]. We use these data in this study to allow comparison with epithelial cells from identical but cryopreserved tissues (Table 1).

### 4.2. Cryopreservation of Nasal Polyp Tissue

Nasal polyp tissues were harvested in endoscopic sinus surgeries. The tissues were briefly washed with povidone-iodine solution (Braunol, Braun, Melsungen, Germany) and rinsed twice with 0.9% sodium chloride. The tissues were stored in Dulbecco’s modified Eagle’s medium (with high glucose) with 10% fetal calf serum and penicillin/streptomycin (final concentration of 100 U/mL and 100 µg/mL) at 4 °C until processing. Freezing work was done within 6 h after surgical removal. The polyp tissues were cut in half or quarters so that the longest dimension did not exceed 1 cm. The tissue was incubated in a cryovial with 1 mL freezing medium (Cryo-SFM, cat #: C-29912, PromoCell, Heidelberg, Germany) for approximately 10 min at room temperature. The cryovials were placed in a freezing chamber (CoolCell, Corning, AZ, USA) and stored at −80 °C for approximately 48 h. Then, the cryovials were stored in liquid nitrogen until they were used.

### 4.3. Isolation of Epithelial Cells from Nasal Polyp Tissue

**Figure 7:**
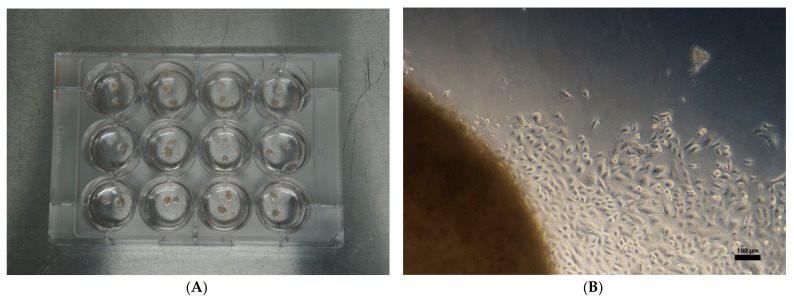
Isolation of epithelial cells using the outgrowth technique. Per sample, 24 small tissue pieces were cultured in a 12-well plate for the isolation of epithelial cells by outgrowth (**A**). Epithelial cell outgrowth (**B**) was evaluated microscopically every 2 days up to 8 days.

Cryopreserved tissues were thawed by incubating in a water bath at 37 °C for approximately 5 min. Epithelial cell isolation was performed using the outgrowth technique described in our previous study [15]. Briefly, tissue was cut into small pieces (1–1.5 mm in diameter), and 24 small tissue pieces per sample were cultured with bronchial epithelial growth medium (BEGM, CC-3170, Lonza, Switzerland) in a 12-well plate (Multidish 12 Wells Nunclon Delta Surface, cat #: 150628, Nunc, Roskilde, Denmark) for the isolation of epithelial cells by outgrowth (Figure 7A). Outgrowth was evaluated microscopically every 2 days, up to 8 days (Figure 7B). To evaluate proliferation, cells were harvested using trypsin-EDTA solution (0.25% and 0.02%, respectively), washed twice with phosphate-buffered saline without calcium and magnesium (PBS), centrifuged at 300× *g*, and transferred to a 6-well plate at 5000 cells per well (total of 30,000 cells). The total number of cells from the 6 wells was counted every 6 days until the 3rd passage.

Isolation of epithelial cells from 3 patients’ tissues after long-term cryopreservation (262, 196, and 191 days, respectively) was demonstrated, and the outgrowth and proliferation up to the 3rd passage were evaluated (Polyps 1–3, see Table 1). In addition, the outgrowth and proliferation of epithelial cells from 3 other fresh tissues and their cryopreserved tissues were compared (Polyps 4–6, see Table 1).

### 4.4. Flow Cytometry

Flow cytometric analysis (using CytoFlex, Beckman Coulter, Munich, Germany) with anti-cytokeratin-PE (Miltenyi Biotec, clone: REA831, Bergisch Gladbach, Germany), anti-p63 (Ventana Medical, clone: 4A4, Tucson, AZ, USA), and anti-Ki-67-FITC (Miltenyi Biotec, clone: REA183, Bergisch Gladbach, Germany) was performed with Polyps 1–3 (Table 1) as described in our previous study to identify epithelial cells and to assess their differentiation and proliferation [15]. Cells from 2nd and 3rd passages were used for the analysis. Cells were cultured in a 75 cm^2^ cell culture flask with 10 mL BEGM until density reached 70-80% confluence, renewing the medium every 2 days. A total of 5 × 10^5^–1 × 10^6^ cells were collected in 300 µL incubation buffer (0.5% bovine serum albumin in PBS). The supernatant was removed after centrifuging at 300× *g* for 10 min at 4 °C, and the cells were washed 3 times in total in the same way by adding 300 µL incubation buffer followed by centrifugation (the same washing process was carried out after each step and will not be mentioned from here on). Cells were fixed in 500 µL of 4% formaldehyde for 15 min at room temperature. After removing the formaldehyde, permeabilization was carried out with 300 µL precooled permeabilization buffer (0.5% Octoxynol-9, 0.68 µM ethylenediaminetetraacetic acid, and 1% bovine serum albumin in distilled aqua) for 10 min on ice. The permeabilization buffer was removed, and cells were incubated first with 300 µL unconjugated anti-p63 (dilution 1:200 in incubation buffer) overnight at 4 °C and then with secondary anti-mouse AlexaFluor633-conjugated antibody (dilution 1:200 in incubation buffer; Molecular Probes, cat #: A-21052, Eugene, OR, USA) for 1 h in a darkened chamber at room temperature. Cells were washed and incubated with fluorescent-dye conjugated anti-cytokeratin-PE and anti-Ki-67-FITC (each dilution 1:200 in incubation buffer) overnight at 4 °C in a darkened chamber. Cells were washed and resuspended in 100 µL incubation buffer to perform a flow cytometric analysis. Compensation was set up before analysis, and positive and negative populations were predefined accordingly.

### 4.5. Enzyme-Linked Immunosorbent Assay 

Epithelial cell function was assessed by the production of typical cytokines of type 2 inflammation, representatively TSLP and periostin. Concentrations of TSLP and periostin in culture supernatants were measured by ELISA according to the manufacturer’s instructions (DY1398-05 and DY3548B R&D Systems, MN, USA) and as described previously [15]. To obtain the supernatants, 5,000 cells were seeded in a 24-well plate with 1 mL BEGM, and the medium was renewed every 2 days until 70–80% confluence. Next, 1 mL of BEGM was added, and supernatants were collected after 24, 48, and 72 h. Cells from the 2nd or 3rd passage of cryopreserved Polyps 3–5 were used for the analysis.

### 4.6. Statistical Analysis

Statistical analysis was performed to compare the outgrowth and proliferation from both fresh tissue and cryopreserved tissue using multiple paired parametric *t*-tests. Data were expressed as mean ± standard error of the mean (SEM). GraphPad Prism 9.4.1 was used for the statistical analysis.

## Figures and Tables

**Table 1:** The demographic and clinical characteristics of tissue donors in this study. The data from Polyps 4–6 were already used in our previous work to demonstrate the isolation of epithelial cells from fresh tissue (named Polyp 1, Polyp 2, and Polyp 3, respectively, in our previous work. Adapted with permission from 2023, Kim et al. Ref. [15]). We use these data in this study to allow a comparison with epithelial cells from identical but cryopreserved tissues.

	Polyp 1	Polyp 2	Polyp 3	Polyp 4	Polyp 5	Polyp 6
Cryopreservation (days)	262	196	191	0 vs. 89	0 vs. 77	0 vs. 75
Age	59	47	58	45	65	45
Sex	Male	Male	Male	Female	Male	Male
Nasal polyp score (right/left)	3/1	3/3	3/3	2/3	3/1	3/4
Prior sinus surgeries	No	No	Yes	No	No	No
Tissue eosinophilia	Yes	Yes	Yes	Yes	Yes	Yes
Asthma	No	No	No	No	No	No
Non-steroidal anti-inflammatory drug exacerbated respiratory disease	Uncertain	No	No	No	No	Yes

## Data Availability

The analyzed datasets generated during the present study are available from the corresponding author upon reasonable request.

## Appendix A

Polyp 4: Out of a total of 24 tissue pieces, outgrowth was noted from 11, 20, 23, and 24 of fresh tissue and 1, 7, 13, and 13 of cryopreserved tissue on days 2, 4, 6, and 8, respectively. On day 8 of outgrowth, a total of 320,000 and 45,000 cells were isolated from fresh tissue and cryopreserved tissue, respectively. The proliferation of both groups up to the 3rd passage was comparable (205,000, 687,000, and 315,000 cells vs. 217,500, 592,500, and 340,000 cells from fresh tissue and cryopreserved tissue at passage 1, 2, and 3, respectively). Polyp 5: Outgrowth of 2, 18, 19, and 19 of fresh tissue and 4, 13, 16, and 17 of cryopreserved tissue out of a total of 24 tissue pieces on days 2, 4, 6, and 8, respectively, was noted. On day 8 of outgrowth, a total of 320,000 and 45,000 cells were isolated from fresh tissue and cryopreserved tissue, respectively. On day 8 of outgrowth, a total of 90,000 and 102,500 cells were isolated from fresh tissue and cryopreserved tissue, respectively. The proliferation rate was much different in the 1st passage but similar in the 2nd and 3rd passages (550,000, 332,500, and 240,000 cells vs. 215,000, 445,000, and 192,500 cells from fresh tissue and cryopreserved tissue at passage 1, 2, and 3, respectively). Polyp 6: Outgrowth was noted from 20, 24, 24, and 24 of fresh tissue and 7, 18, 23, and 23 of cryopreserved tissue out of a total of 24 tissue pieces on days 2, 4, 6, and 8, respectively. On day 8 of outgrowth, a total of 320,000 and 45,000 cells were isolated from fresh tissue and cryopreserved tissue, respectively. On day 8 of outgrowth, a total of 320,000 and 160,000 cells were isolated from fresh tissue and cryopreserved tissue, respectively. The proliferation rate showed a similar trend but with different fold change (400,000, 545,000, and 375,000 cells vs. 180,000, 657,500, and 117,500 cells from fresh tissue and cryopreserved tissue at passage 1, 2, and 3, respectively).
